# Pilot study quantifying muscle glycosaminoglycan using bi-exponential T_1ρ_ mapping in patients with muscle stiffness after stroke

**DOI:** 10.1038/s41598-021-93304-7

**Published:** 2021-07-06

**Authors:** Rajiv G. Menon, Preeti Raghavan, Ravinder R. Regatte

**Affiliations:** 1 Bernard and Irene Schwartz Center for Biomedical Imaging, New York University School of Medicine, 660 1st Ave, 4th Floor, New York, NY 10016 USA; 2 Deparments of Physical Medicine and Rehabilitation and Neurology, School of Medicine, Johns Hopkins University, Baltimore, MD USA

**Keywords:** Prognostic markers, Imaging techniques

## Abstract

Post stroke muscle stiffness is a common problem, which left untreated can lead to disabling muscle contractures. The purpose of this pilot study was to evaluate the feasibility of bi-exponential T_1ρ_ mapping in patients with arm muscle stiffness after stroke and its ability to measure treatment related changes in muscle glycosaminoglycans (GAGs). Five patients with muscle stiffness after stroke and 5 healthy controls were recruited for imaging of the upper arm with 3D-T_1ρ_ mapping. Patients were scanned before and after treatment with hyaluronidase injections, whereas the controls were scanned once. Wilcoxon Mann–Whitney tests compared patients vs. controls and patients pre-treatment vs. post-treatment. With bi-exponential modeling, the long component, T_1ρl_ was significantly longer in the patients (biceps *P* = 0.01; triceps *P* = 0.004) compared to controls. There was also a significant difference in the signal fractions of the long and short components (biceps *P* = 0.03, triceps *P* = 0.04). The results suggest that muscle stiffness is characterized by increased muscle free water and GAG content. Post-treatment, the T_1ρ_ parameters shifted toward control values. This pilot study demonstrates the application of bi-exponential T_1ρ_ mapping as a marker for GAG content in muscle and as a potential treatment monitoring tool for patients with muscle stiffness after stroke.

## Introduction

Following a neurological injury, such as a stroke, individuals can develop severe muscle stiffness, which contributes to muscle fatigue, lack of control of movement, pain, disability, and eventually to permanent soft tissue contractures^1,2^. While spasticity results from reduced cortical influence and subsequent disinhibition of spinal cord stretch reflexes, causing hyperreflexia, peripheral changes in the muscle also contribute to muscle stiffness, and greatly impacts quality of life and the long-term cost of care^3,4^. Spasticity and muscle stiffness occur in several neurological conditions including multiple sclerosis (MS), cerebral palsy, traumatic brain injury (TBI) and spinal cord injury^5–8^, but the underlying mechanisms are largely unknown. The hyaluronan hypothesis postulated that the deposition of hyaluronan in the extra-cellular matrix (ECM) contributes to the development of muscle stiffness^9^. Hyaluronan is a high molecular weight glycosaminoglycan (GAG), a chief component of the ECM in all tissues including muscle where it serves as a lubricant, allowing contracting muscle fibers to glide past each other to transmit forces^10,11^. Studies have shown that muscle disuse causes excessive deposition of hyaluronan in the ECM, leading to a phase change in its properties; aggregation of excess hyaluronan dramatically increases the viscosity and stiffness of the ECM^12–14^. Until recently, treatment options to address muscle stiffness have focused on suppressing spasticity or muscle overactivity using central nervous system (CNS) depressants, and directly reducing muscle activity using nerve blocks and botulinum toxin injections. However, these do not address the physical changes occurring in the muscle itself that may produce stiffness^15,16^.

Raghavan et al.^9^ previously showed that off-label intramuscular injections of the enzyme hyaluronidase, which hydrolyzes the excess aggregated hyaluronan, was safe and well tolerated in patients with severe upper limb muscle stiffness. It led to significantly reduced stiffness and improved range of motion post-injection, but structural changes in the muscle were not quantified. Skeletal muscle is a heterogeneous mix of tissue components, such as collagen, muscle fiber, fat, and extracellular matrix components such as GAGs. T_1ρ_ mapping is an MRI technique that is sensitive to low energy interactions related to the chemical exchange between extracellular water and complex macromolecules and has been used to quantify GAG content in articular cartilage^17–20^. In recent studies we showed that mono-exponential T_1ρ_ mapping can be used to monitor treatment response in patients with muscle stiffness^21,22^. However, mono-exponential T_1ρ_ is a macro measurement of an extremely complex microenvironment in the ECM of the muscle. A number of studies have suggested that bi-exponential T_1ρ_ mapping may better represent the diverse microenvironment that gives rise to the relaxation times in tissue types such as cartilage^23–25^, brain^26,27^, liver^28^ as well as skeletal muscle^29^. There have been no studies on the application of multi-exponential T_1ρ_ mapping for the measurement of the muscle microenvironment in muscle stiffness. A multi-exponential T_1ρ_ mapping measurement model can quantify the fractions and components that better represent the nature of changes occurring in the microenvironment of the muscle to better understand the mechanisms underlying this novel treatment, and potentially be useful to monitor the treatment response.

In this study, we evaluate the feasibility of using bi-exponential modeling of T_1ρ_ mapping to quantify GAG levels in patients with post-stroke muscle stiffness before and after hyaluronidase injection treatments compared to a control cohort.

## Results

Five post-stroke patients were enrolled in the study and scanned pre-treatment. Three of the five patients returned for the post-treatment MRI scan at least two weeks after hyaluronidase injections. Five control subjects enrolled in the study underwent MRI scans once.

### Controls versus patients

**Figure 1:**
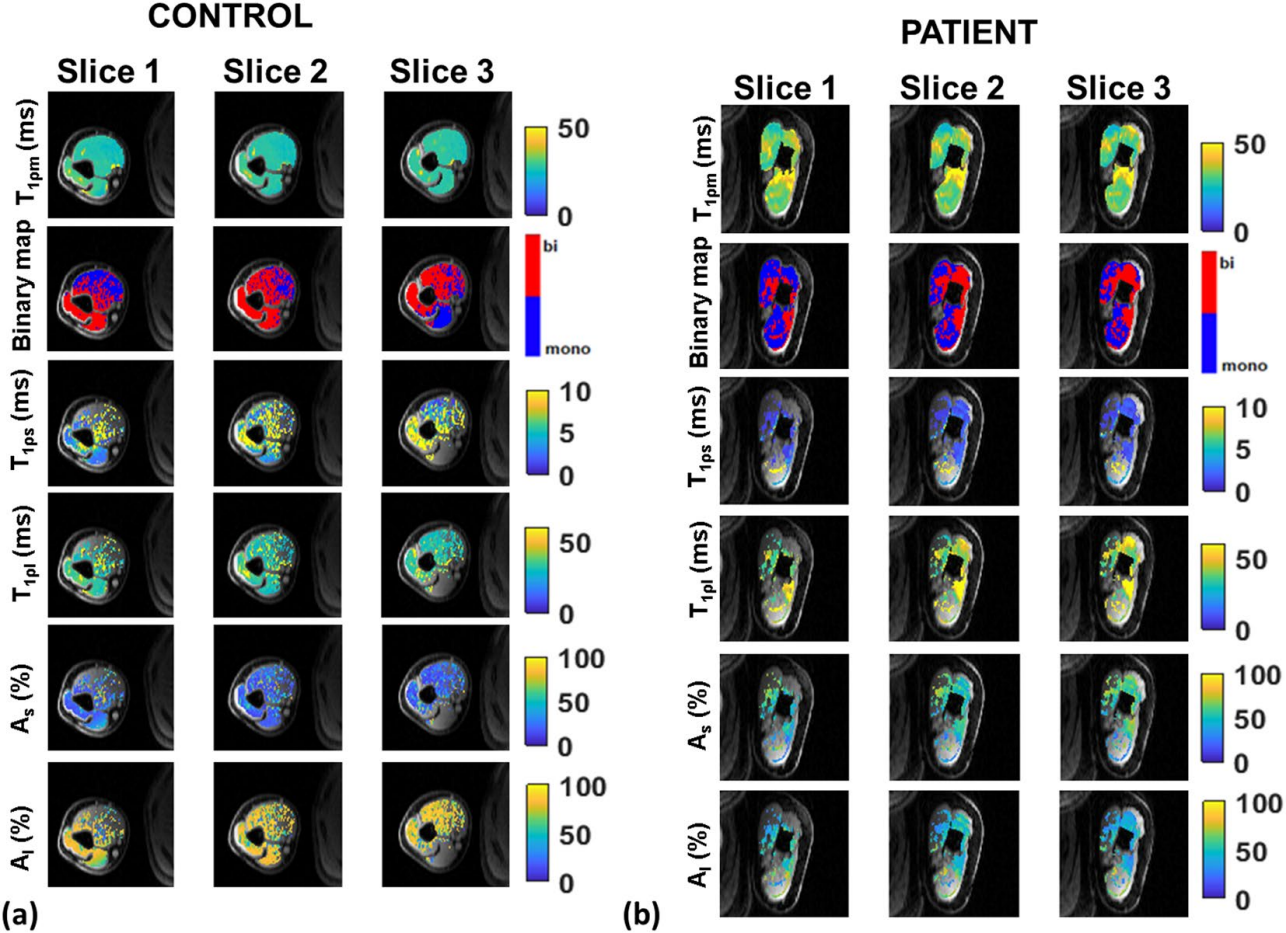
Bi-exponential results in control subject and patient. (**a**) shows mono-exponential (T_1ρm_) maps and bi-exponential maps (binary map, T_1ρs_, and T_1ρl_) in a control subject and (**b**) shows the results obtained from the affected arm of a patient with post stroke muscle stiffness before treatment. T_1ρm_ (row 1) in the patient is significantly higher compared to the control, while T_1ρs_ values (row 3) are shorter, and T_1ρl_ (row 4) are longer than in the control subject.

Figure 1a shows representative T_1ρ_ maps in three slices from a control subject overlaid over the corresponding anatomical slices, and Fig. 1b shows similar T_1ρ_ maps in a representative patient with post stroke muscle stiffness (before treatment). The first row shows mono-exponential T_1ρ_ maps in the control subject and the patient. Note that the mono-exponential relaxation times are longer in the patient. The second row shows a binary map of mono-exponential or bi-exponential pixels in each slice. The third row shows the short T_1ρ_ relaxation map, T_1ρs._ Although the T_1ρs_ in this control subject is longer in the biceps and the triceps compared to the patient, a significant difference was not observed across the control and patient groups pre-treatment. The fourth row of images shows the long component of the T_1ρ_ relaxation map, T_1ρl_ which was longer in the patient. The mean T_1ρl_ across all controls was 37.32 ± 2.01 ms in the biceps and 38.99 ± 4.89 ms in the triceps. In comparison, the mean T_1ρl_ was significantly longer in the patients (biceps = 46.33 ± 5.57 ms, *P* = 0.01 and triceps = 50.5 ± 9.25 ms, *P* = 0.004). The fifth and sixth rows show the signal fractions for the short component (A_s_ (%)) and the long component (A_l_ (%)). Between controls and patients there is also a significant difference in the relative signal fraction contributions to the long and short components, the fraction of the short component being significantly larger in patients compared to controls pre-treatment (*P* = 0.03 in biceps, and *P* = 0.04 in triceps). The data of the mono- and bi-exponential analysis in the two cohorts is summarized in Table 1.

**Table 1:** Summary of mono-exponential and bi-exponential T_1ρ_ mapping results.

ROI	Fitting method	Parameter	Controls (n = 5)	Patients-PRE (n = 5)	Patients-POST (n = 3)	*P* values		
						Patients vs controls	Pre- vs post-injection	Post-injection vs controls
Biceps	Mono-exponential	T_1ρm_ (ms)	26.7 ± 0.54	35.5 ± 2.93	29.45 ± 1.23	0.006	0.055	0.660
	Bi-exponential	T_1ρs_ (ms)	7.12 ± 1.97	4.16 ± 3.25	7.72 ± 3.28	0.490	0.510	0.890
		T_1ρl_ (ms)	37.32 ± 2.01	46.33 ± 5.57	48.14 ± 7.19	0.010	0.820	0.011
		A_s_ (%)	31.68 ± 8.54	52.21 ± 14.43	42.53 ± 9.17	0.031	0.410	0.070
		A_l_ (%)	68.32 ± 8.54	47.79 ± 14.43	57.47 ± 9.17	0.031	0.410	0.070
Triceps	Mono-exponential	T_1ρm_ (ms)	30.29 ± 2.23	34.57 ± 4.48	32.91 ± 4.9	0.138	0.730	0.500
	Bi-exponential	T_1ρs_ (ms)	8.02 ± 3.26	6.06 ± 4.45	6.84 ± 1.40	0.930	0.90	0.460
		T_1ρl_ (ms)	38.99 ± 4.89	50.5 ± 9.25	44.12 ± 6.13	0.004	0.280	0.150
		A_s_ (%)	28.06 ± 7.32	52.66 ± 20.29	42.54 ± 23.00	0.040	0.450	0.190
		A_l_ (%)	71.94 ± 7.32	47.34 ± 20.29	57.46 ± 20.29	0.040	0.450	0.190

Mean T_1ρ_ values (± SD) for mono-exponential (T_1ρm_) and bi-exponential (T_1ρs_, T_1ρl_, A_s_, A_l_) results in the biceps and triceps ROIs.

### Patient pre- versus post-treatment

**Figure 2:**
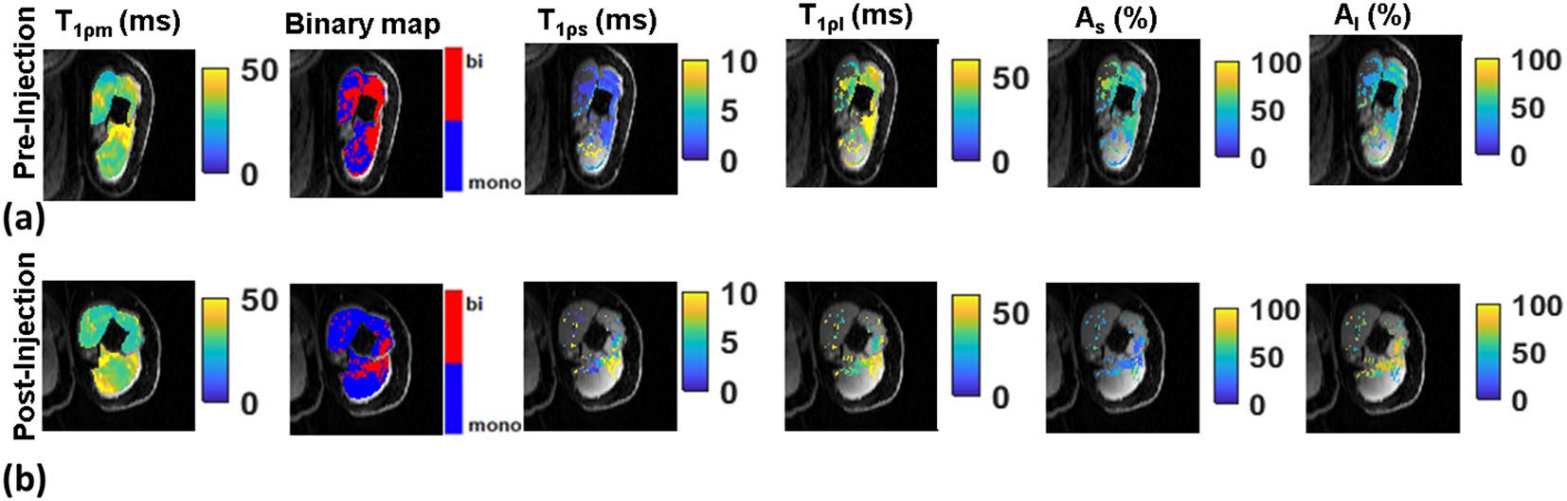
Bi-exponential T_1ρ_ mapping pre- and post-injection treatment. (**a**) shows the results in a patient pre-treatment (**b**) shows the same patient following hyaluronidase injections at approximately the same slices. Note changes in the shape of the muscle. In the biceps, note considerable reduction in T_1ρm_ values, and shift of T_1ρs_ and T_1ρl_ towards normative values.

Figure 2 shows representative results of mono- and bi-exponential T_1ρ_ maps before and after hyaluronidase injection treatment in patients with post stroke muscle stiffness. In the figure, slices from similar locations are shown pre- and post-treatment. Changes in the shape of the muscles are apparent following treatment. Considerable improvements in the T_1ρ_ values are seen in the patient cohort from pre- to post-treatment. While the pre and post treatment changes do not reach statistical significance (Table 1), the post-injection values approach those of controls.

**Figure 3:**
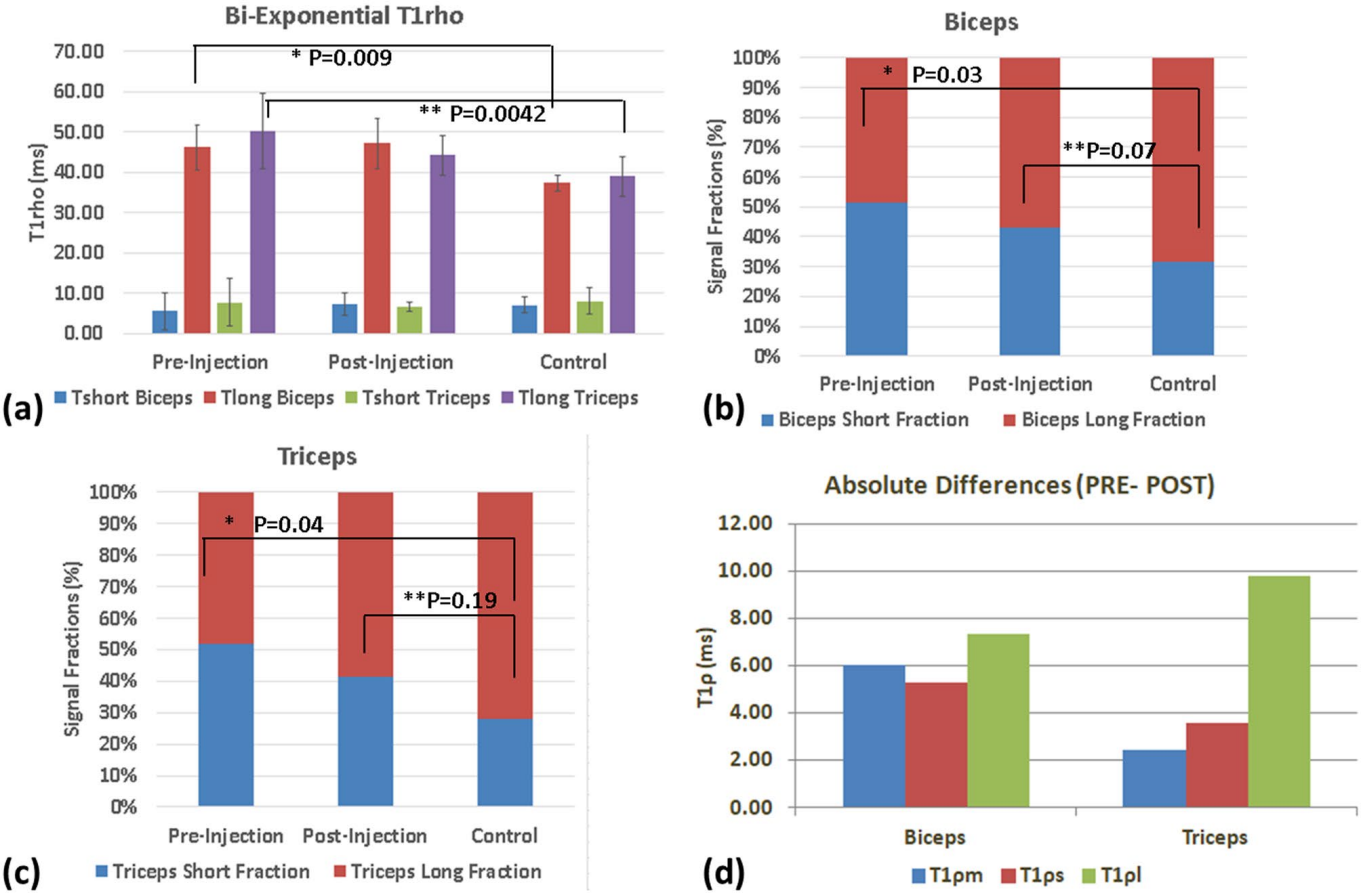
Comparison of mean T_1ρ_ values between groups. (**a**) shows bi-exponential results in biceps and triceps ROIs between patients pre- and post-treatment and controls, (**b**) shows the comparison of the signal fractions in the biceps between pre- and post-treatment and controls, (**c**) shows the comparison of the signal fractions in the triceps between pre- and post-treatment, and controls, and (**d**) shows absolute differences in T_1ρm_, T_1ρs_, T_1ρl_ between pre- and post-treatment in the biceps and triceps.

Figure 3a shows the comparison of bi-exponential T_1ρ_ mapping between the cohorts demonstrating that additional information can be gleaned from the bi-exponential analysis of the data. A significant difference is observed between controls and patients pre-treatment in the long T_1ρ_ component for both biceps (*P* = 0.01) and triceps (*P* = 0.004) muscles. Post-treatment there was a significant change in the biceps signal fraction (*P* = 0.03) (Fig. 3b), and in the triceps signal fraction (*P* = 0.04) (Fig. 3c). Note that post treatment, there was no statistically significant difference between the patient and control cohorts. Figure 3d shows the absolute differences between the pre- and post-treatment cohort for T_1ρm,_ T_1ρs,_ and T_1ρl_ for the bicep and tricep ROIs. The biggest change was seen in the long component post-treatment in both ROIs.

### Model of GAG changes and measurements made using bi-exponential T1ρ

**Figure 4:**
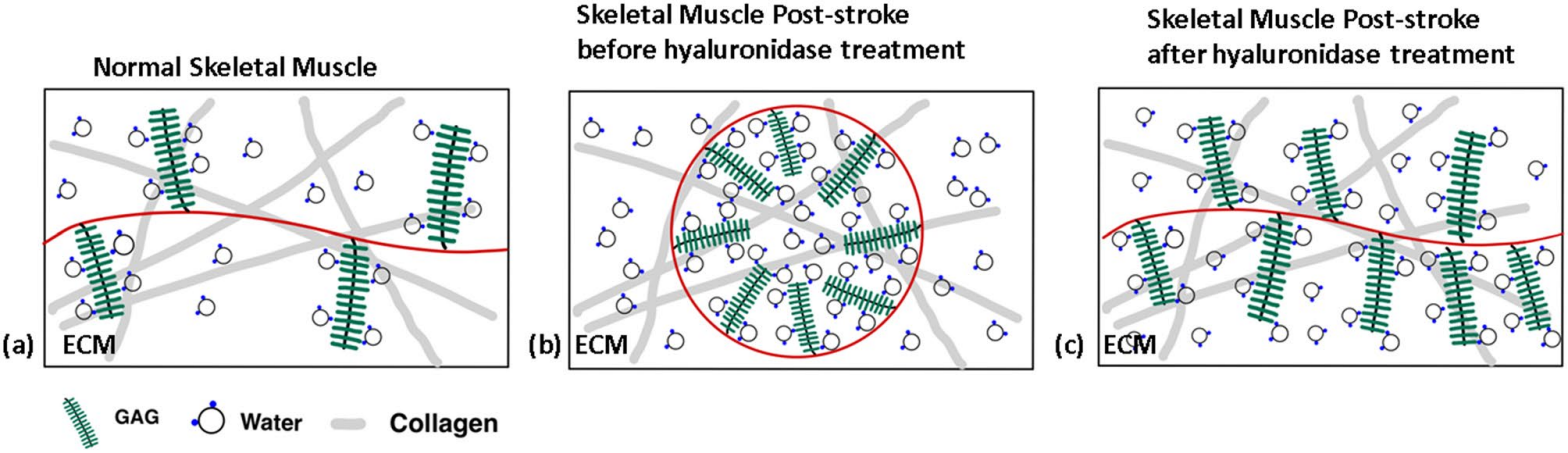
Schematic model of changes in GAG content and structure and its measurements with bi-exponential T_1ρ._ (**a**) shows a cross-section of the molecular environment in normal skeletal muscle. T1ρ measures the chemical exchange between negatively charged GAG and protons in water. In (**b**), excessive GAG in stiff muscles undergoes a phase change, forming aggregates and trapping water in macromolecular spheres. The relative fraction of the short component increases due to GAG accumulation. T_1ρs_ is relatively unchanged as the chemical exchange is unchanged, but T_1ρl_ increases due to the trapped free water. In (**c**), treatment with hyaluronidase breaks down the GAG aggregates/ macromolecular spheres, with a shift in T_1ρ_ parameters to more normal values.

Figure 4 shows a schematic representation of the molecular changes in skeletal muscle ECM to explain the measurements made using bi-exponential T_1ρ_. Figure 4a shows the microenvironment in normal skeletal muscle, where T_1ρ_ measurements reflect the chemical exchange between negatively charged GAG molecules and the surrounding protons in the water molecules. Figure 4b shows possible changes in the microenvironment of stiff muscles. Aggregation of hyaluronan causes a phase change, forming hyaluronan macromolecular spheres that trap free water^14^. The measured relative signal fraction of the short component increases, but our data shows that T_1ρs_ does not change much relative to controls, as the chemical exchange is unchanged. The measured T_1ρl_ increases due to an increased proportion of water molecules that are trapped and cannot participate in the chemical exchange. Figure 4c shows the change following hyaluronidase treatment, which breaks down the GAG aggregates and releases the trapped water. The measured T_1ρ_ short fraction and T_1ρl_ move towards normal values.

## Discussion

This study demonstrates the application of bi-exponential T_1ρ_ mapping as a surrogate marker of changes in GAG content and structure in a cohort of patients with post stroke muscle stiffness. This study further characterizes the effect of hyaluronidase injections in skeletal muscle using mono- and bi-exponential T_1ρ_ mapping.

Each voxel shown in the MR image is the aggregate value of protons representing a multi-exponential complex microenvironment. Existence of multiple relaxation components have been reported in multi-component relaxation MRI studies using T_2_ and T_1ρ_ imaging^29–31^. Atleast three components have been identified related to macromolecules, intra- and extra-cellular water, respectively. Normal skeletal muscle is composed of intra-cellular water and extra-cellular water (~ 75%), contractile proteins (~ 20%), and other components (~ 5%) such as salts, phosphates, ions, glycogen, and macronutrients. The short component is a fast-relaxing component that includes tightly bound macromolecules (e.g. collagen, proteoglycans, contractile proteins, and other components), whereas the long component mainly corresponds to loosely bound water (e.g. inflammation and edema).

By using a two-compartment model with T_1ρ_ mapping, one representing the proton chemical exchange with GAGs at fast relaxation rates (T_1ρs_) and the other representing proton exchange in free water at slow relaxation rates (T_1ρl_), we can delineate a clearer picture of the extracellular environment of the muscle. There are few reports of bi-exponential modeling of T_1ρ_ mapping in skeletal muscle. Sharafi et al.^29^ demonstrated the feasibility of bi-exponential mapping in skeletal calf muscles in healthy volunteers. Our results for the control subjects are in agreement with the findings of this study. Yuan et al. ^32^ demonstrated bi-exponential analysis in rat muscles. The mean mono, short and long T_1ρ_ values reported are slightly higher than those reported in our study but are in general agreement. A prior study protocol required the acquisition of 10 different spin lock durations at a higher voxel resolution ranging (1 × 1 mm^2^) from 2 to 55 ms. Acquiring data from ten spin lock times is time consuming (~ 30 min) and challenging to achieve in patients with muscle stiffness. Therefore, we reduced the voxel resolution to 2 × 2 mm^2^ in-plane to reduce the total acquisition time to 10 min, which is more clinically feasible in patient populations. In the future, higher resolution maps may be acquired using prospective compressed sensing techniques^33^.

As observed in other studies with mono-exponential T_1ρ_ analysis, an increase in T_1ρ_ values is observed with increases in GAG content in the skeletal muscle. Bi-exponential T_1ρ_ mapping can provide a more detailed characterization of the changes in the extracellular microenvironment (e.g. chemical exchange rate of protons, pH, GAG concentration, viscosity and presence of free water etc.) that leads to the net increase observed in the mono-exponential T_1ρ_ values. The bi-exponential modeling uses a two-compartment model that breaks down the observed mono-exponential decay into its constituent parts, a short component that relaxes quickly and a long component that relaxes slowly. As shown in Fig. 4, increased GAG accumulation can increase the chemical exchange of the negatively charged GAGs with the protons of the water molecules and increase the fraction of the short component (A_s_ (%)). However, a more significant consequence is the aggregation of the GAGs into macromolecular spheres that trap free water in the ECM and increase the duration of the long component, T_1ρl._ In our study, for the patients with muscle stiffness (pre-treatment), a net increase in the mono-exponential T_1ρ_ suggests increased GAG accumulation as proposed by the hyaluronan hypothesis. From the bi-exponential T_1ρ_ mapping, a closer look at the short and long components and their relative fractions suggests that in patients with stiffness, the relaxation time of the short component is relatively unchanged, but the fraction of the short component increases due to increased GAG accumulation. In contrast, the relaxation time of the long component increases due to excessive free water in the ECM.

Following treatment, we note the shift of the mono-exponential, short component and long components with their corresponding fractions to values approaching those in controls. The changes in the fraction of the short component and the relaxation times of the long component provide an understanding of the mechanisms by which hyaluronidase injections address muscle stiffness. Separate from subjective patient responses, quantitative T_1ρ_ mapping enables objective measurements that can be useful in comparing across subjects and potentially monitoring the dose–response relationship. For example, higher doses of hyaluronidase may be required for a statistically significant pre-post effect, but this may vary based on patient age and sex^34^. Further studies with large cohorts need to be performed to validate this technique as an effective treatment-monitoring tool. Additionally, it may be possible that other systemic processes such as inflammation also contribute to T_1ρ_ measurements, which were not investigated in this study.

Aggregated hyaluronan in muscle is a potential therapeutic target for the treatment of muscle stiffness. According to the hyaluronan hypothesis, muscle stiffness arises as a consequence of disturbed skeletal muscle homeostasis, which maintains a balance between the production and adequate clearance of hyaluronan^10^. Following neurologic injury such as a stroke, paralysis and the ensuing immobility leads to dysregulated hyaluronan homeostasis resulting in its aggregation in the ECM over time. The non-Newtonian properties of hyaluronan lead to dramatic changes in its viscoelastic properties, and a loss in its lubricant properties in the muscle microenvironment, due to which skeletal muscle fibers are no longer able to glide past each other. This process may be exacerbated due to the loss of neural control and neural hyper-excitability, which may lead to increased stimulation of the fasciacytes that produce hyaluronan^35^, further contributing to its accumulation, aggregation and muscle stiffness. In addition, disuse causes muscle fibers to atrophy, and a combination of inflammatory, molecular and cellular processes may initiate fibrosis^36,37^. As immobility, muscle atrophy and inflammation continue long term, collagen accumulation in the ECM increases, leading to thickening of the endomysium and perimysium and eventually to permanent fibrosis^38^.

Recent work by Sharafi et al. reported the detection of liver fibrosis using bi-exponential T_1ρ_ mapping^28^. Mono-exponential T_1ρ_ mapping is useful, cannot distinguish between the major components of pathology such as inflammation/edema and fibrogenic activity. While more studies are needed to prove a definitive relationship, it is reasonable to suggest that the bi-exponential short component is related to macromolecules, proteoglycans and collagens that contribute to fibrogenic activity, whereas the long component is related to processes that increase free water such as inflammation and edema. Such delineation between fibrosis and inflammation is not validated yet in patients with muscle stiffness using independent reference standards. In patients post stroke, we expect to see a change in the short T_1ρ_ component in patients with fibrosis due to connective tissue accumulation and a change in the long component due to inflammation and edema. The bi-exponential T_1ρ_ mapping represents a non-invasive surrogate marker to quantify GAG content and structure in individuals with muscle stiffness. We speculate that the stage of muscle stiffness prior to the development of permanent contracture may be an opportune window for therapeutic intervention to hydrolyze the aggregated hyaluronan, restore hyaluronan homeostasis in skeletal muscle, and prevent fibrosis. Intramuscular hyaluronidase injections hydrolyze the aggregated hyaluronan, reducing stiffness without exacerbating muscle weakness, thereby enabling increased mobility to potentially reverse the progression to muscle contracture^9^. Mono and bi-exponential T_1ρ_ mapping can be helpful in quantifying GAG content and structure, establishing the mechanism underlying muscle stiffness, and assist in potentially informing the dose–response relationship during treatment. Further studies are required to investigate the clinical utility of the therapy and the imaging. Additionally, these methods can be easily extended to other causes of muscle stiffness, where the pattern of hyaluronan accumulation may be different ^22^.

This study had some limitations—the patient study cohort was small. While this study only seeks to demonstrate feasibility of bi-exponential T_1ρ_ mapping, a larger study with a diverse patient population before and after treatment would be needed to establish the clinical utility of this technique. The control arm of the study was not age and sex matched and does not take into account the effect of aging and sex on T_1ρ_ values in skeletal muscle^34^.

In summary, this pilot study demonstrates the feasibility of bi-exponential T_1ρ_ mapping as a quantitative marker of GAG content and structure in patient populations that experience muscle stiffness. This study characterizes the effect of hyaluronidase injections in skeletal muscle using mono- and bi-exponential T_1ρ_ mapping and may be of potential use as a diagnostic and/or treatment monitoring tool.

## Materials and methods

### Study design

This prospective study was approved by the New York University institutional review board (IRB), was health information portability and accountability act (HIPAA) compliant and all methods were performed in accordance with the IRB guidelines and regulations. All recruited patients and healthy volunteers provided written informed consent.

### Subjects

Two cohorts were recruited for the study. The post-stroke cohort included patients who were adults over 18 years that had suffered a stroke and experienced severe muscle stiffness and were receiving off-label intramuscular hyaluronidase injections (Hylenex, Halozyme Therapeutics, Inc) as part of their clinical treatment. Five patients were recruited (2 males/3 females, age = 52 ± 3 years). For the control cohort, healthy adult volunteers were recruited and scanned once using the same MR imaging protocol used for the patients. A total of five healthy volunteers were recruited (1 male/ 4 females, age = 27 ± 2 years).

### Study protocol

For this imaging study, the patients underwent pre-treatment MRI scans before the hyaluronidase injections, and post-treatment MRI at least two weeks after a single treatment. The intramuscular injection doses and locations of treatment were similar to those reported in a previous publication^9^, and were provided in several muscles based on each patient’s clinical presentation, including those imaged in this study.

All imaging scans were performed on clinical 3T MR scanner (Prisma, Siemens Healthineers, Erlangen, Germany). The scanner body coil was used for transmit (Tx) and a flexible 8-array receive coil (Rx) was wrapped around the upper arm and placed as close to the center of the bore as possible. The imaging protocol consisted of multiple spin-lock, 3D-T_1ρ_ mapping scans. For T_1ρ_ mapping a 3D Cartesian turbo-FLASH sequence was used, preceded by a customized T_1ρ_ preparation module used with different spin-lock durations, and followed by a delay for T_1_ restoration. A paired self-compensated spin-lock pulse was used to minimize B_0_ and B_1_ variations. The sequence parameters included FOV = 130 mm, matrix size of 256 × 64 × 64, giving a voxel resolution of 0.5 × 2 × 2 mm^3^. TR = 7.5 ms, TE = 4 ms, spin lock frequency was 500 Hz, ten TSLs including 2, 4, 6, 8, 10, 15, 25, 35, 45, and 55 ms. The total acquisition time was about 10 min.

### Modeling and data analysis

**Figure 5:**
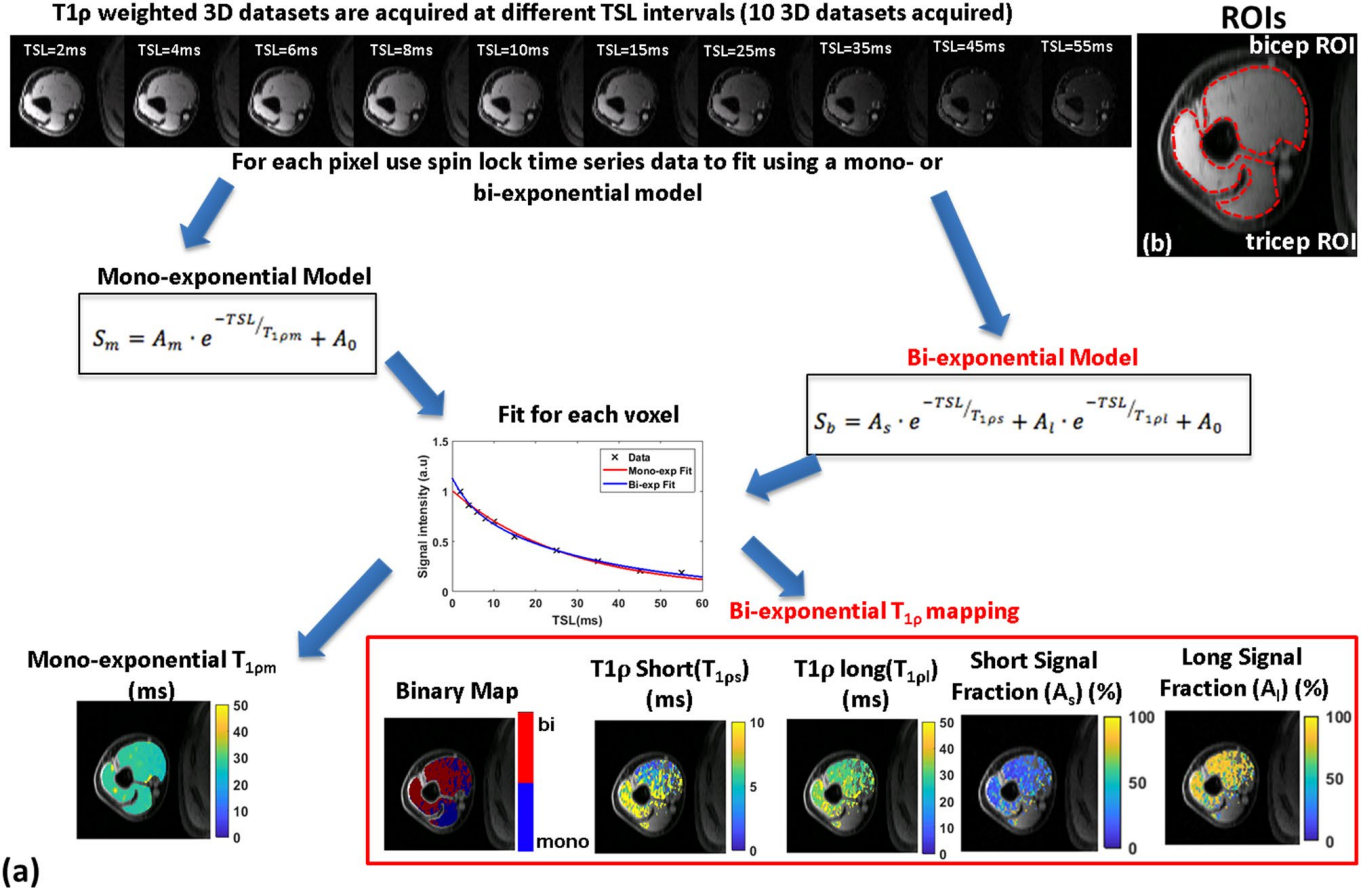
Post-processing pipeline for T_1ρ_ mapping. (**a**) shows a schematic representation of the T_1ρ_ mapping pipeline. T_1ρ_ weighted image time series data acquired at a range of spin-lock times are used. Mono-exponential modeling uses a three parameter fit producing T_1ρm_ parameter map. Bi-exponential model uses the same data and uses a five parameter fit to perform the modeling to generate a binary map showing voxels displaying mono- or bi-exponential relaxation, T_1ρs_, T_1ρl_, and relative signal fractions (A_s_ and A_l_). The ROIs used for the biceps and triceps are shown in (**b**).

Figure 5a shows the schematic representation of data modeled and analyzed for bi-exponential T_1ρ_ mapping. The time-series data for each spin lock time from 2 to 55 ms for each pixel were used for fitting. Manual regions of interest (ROIs) were created as binary masks for the biceps and triceps muscle groups (Fig. 5b). Mono-exponential T_1ρ_ mapping was performed by fitting the signal intensity across the TSLs for each pixel as reported previously^21^.

The bi-exponential modeling was performed using the same data as mono-exponential modeling. Here the fitting was performed as components of two separate relaxation components within each voxel using a five-parameter non-linear bi-exponential model, as shown in Eq. (1):

1 $$S = A_{s} \cdot e^{{{\raise0.7ex\hbox{${ - TSL}$} \!\mathord{\left/ {\vphantom {{ - TSL} {T_{{1\rho s}} }}}\right.\kern-\nulldelimiterspace} \!\lower0.7ex\hbox{${T_{{1\rho s}} }$}}}} + A_{l} \cdot e^{{{\raise0.7ex\hbox{${ - TSL}$} \!\mathord{\left/ {\vphantom {{ - TSL} {T_{{1\rho l}} }}}\right.\kern-\nulldelimiterspace} \!\lower0.7ex\hbox{${T_{{1\rho l}} }$}}}} + A_{0}$$

where, A_s_ is the fraction of the signal contributing to the fast relaxing component, and A_l_ is the fraction of the signal contributing to the slower relaxing component. T_1ρs_ is the short component T_1ρ_ relaxation time, and T_1ρl_ is the long T_1ρ_ relaxation component. The bi-exponential T_1ρ_ fitting starts with fitting mono-exponential results and classifying them as either short components (range = 0–10 ms) or long components (range = 10–200 ms). The F-test was used with a criterion set to F-ratio > 5.14 to distinguish between mono-and bi-exponential voxels^32^. The bi-exponential binary maps were calculated for every voxel in the ROI using a 3 × 3 averaging filter. To estimate the standard deviation of the noise and signal to noise ratio (SNR), the Marchenko-Pastur principal component analysis (MP-PCA) method was used as reported previously^33,39^.

### Statistics

Mean values and standard deviations were calculated for each of the ROIs drawn for each cohort. Wilcoxon Mann–Whitney test was used to compare the pre vs. post treatment in the patient cohort, and the patient vs. control cohorts. A *P* value of less than 0.05 was used as the threshold to reject the null hypothesis.
